# The Quality of Reporting Methods and Results in Network Meta-Analyses: An Overview of Reviews and Suggestions for Improvement

**DOI:** 10.1371/journal.pone.0092508

**Published:** 2014-03-26

**Authors:** Brian Hutton, Georgia Salanti, Anna Chaimani, Deborah M. Caldwell, Chris Schmid, Kristian Thorlund, Edward Mills, Ferrán Catalá-López, Lucy Turner, Douglas G. Altman, David Moher

**Affiliations:** 1 Ottawa Hospital Research Institute, Ottawa, Ontario, Canada; 2 Faculty of Medicine, University of Ottawa, Ottawa, Ontario, Canada; 3 Department of Hygiene and Epidemiology, School of Medicine, University of Ioannina, Ioannina, Greece; 4 School of Social and Community Medicine, University of Bristol, Bristol, United Kingdom; 5 Center for Evidence-based Medicine, Program in Public Health, Brown University, Providence, Rhode Island, United States of America; 6 Faculty of Health Sciences, University of Ottawa, Ottawa, Ontario, Canada; 7 Standard Prevention Research Center, Stanford University, Stanford, California, United States of America; 8 Clinical Epidemiology and Biostatistics, McMaster University, Hamilton, Ontario, Canada; 9 Division of Pharmacoepidemiology and Pharmacovigilance, Spanish Medicines and Healthcare Products Agency (AEMPS), Madrid, Spain; 10 Oxford Centre for Statistics in Medicine, University of Oxford, Oxford, United States of America; Canadian Agency for Drugs and Technologies in Health, Canada

## Abstract

**Introduction:**

Some have suggested the quality of reporting of network meta-analyses (a technique used to synthesize information to compare multiple interventions) is sub-optimal. We sought to review information addressing this claim.

**Objective:**

To conduct an overview of existing evaluations of quality of reporting in network meta-analyses and indirect treatment comparisons, and to compile a list of topics which may require detailed reporting guidance to enhance future reporting quality.

**Methods:**

An electronic search of Medline and the Cochrane Registry of methodologic studies (January 2004–August 2013) was performed by an information specialist. Studies describing findings from quality of reporting assessments were sought. Screening of abstracts and full texts was performed by two team members. Descriptors related to all aspects of reporting a network meta-analysis were summarized.

**Results:**

We included eight reports exploring the quality of reporting of network meta-analyses. From past reviews, authors found several aspects of network meta-analyses were inadequately reported, including primary information about literature searching, study selection, and risk of bias evaluations; statement of the underlying assumptions for network meta-analysis, as well as efforts to verify their validity; details of statistical models used for analyses (including information for both Bayesian and Frequentist approaches); completeness of reporting of findings; and approaches for summarizing probability measures as additional important considerations.

**Conclusions:**

While few studies were identified, several deficiencies in the current reporting of network meta-analyses were observed. These findings reinforce the need to develop reporting guidance for network meta-analyses. Findings from this review will be used to guide next steps in the development of reporting guidance for network meta-analysis in the format of an extension of the PRISMA (Preferred Reporting Items for Systematic reviews and Meta-Analysis) Statement.

## Introduction

Systematic reviews incorporating meta-analyses (SRMA) have long been used to derive summary comparison measures from multiple sources of evidence, most commonly randomized clinical trials (RCTs), to establish the effectiveness and safety of one treatment relative to another. This approach to evidence synthesis is now considered a standard in evidence based medicine. SRMAs have long been considered a scientifically rigorous means of comparing pairs of different medical interventions. To maximize their transparency, methodologic quality and consistency of reporting, the Quality of Reporting of Meta-Analyses (QUOROM) checklist for authors was developed in 1999 [1]. In 2009, the Preferred Reporting Items for Systematic reviews and Meta-Analysis (PRISMA) statement was developed as a robust update to QUOROM to cover subsequently noted items that were considered essential. [2].

Over time, increasingly large numbers of treatments for many medical conditions have provided clinicians with more choices from which to select a treatment strategy for their patients. Regulators have only required evidence of benefit over no treatment and a lack of evidence of harms for approval to market. The resulting absence of motivation for drug developers to compare their products against those of their competitors has promoted analytic methods to establish the relative benefits of new agents relative to existing therapies. Following work by Higgins and Whitehead [3] in 1996, Bucher et al [4] in 1998 proposed the adjusted indirect comparison, and in subsequent years Lumley [5] (2003) and Lu and Ades [6] (2004) described methods for *network meta-analysis* (related terms including *mixed treatment comparisons meta-analysis* or *multiple treatments meta-analysis*) to simultaneously compare and rank a network of treatments, subsets of which have been compared in individual studies.

The frequency of use of network meta-analysis has risen notably since the mid 2000s, [7]–[9] as has the number of publications addressing methodology for conducting indirect comparisons and network meta-analyses, thereby challenging researchers to keep their approaches to indirect comparisons up to date. This rapid evolution of methods has raised concerns that the conduct and reporting of network meta-analyses, while improving, may not yet be at a sufficiently strong level. Recent guidance documents from the International Society for Pharmacoeconomics and Outcomes Research [10], [11] (ISPOR) and the National Institute for Clinical Excellence [12] (NICE) have attempted to lay out the key ideas for properly implementing these methods.

The PRISMA statement [2] was developed to provide systematic reviewers with guidance on elements to produce optimal reporting of systematic reviews and meta-analyses of pairwise treatment comparisons in order to maximize the transparency, replicability, and quality of such studies. Compared to pairwise comparisons of two treatments, network meta-analysis requires more complex meta-analytic techniques that are associated with additional assumptions, a more complex statistical framework, and generates additional outputs of potential interest to readers (for example, treatment ranks and corresponding probabilities) which can complicate the presentation of results. [13] While documents providing guidance for systematic reviewers and readers on conduct and interpretation of network meta-analysis are available, a guidance document for reporting may also be beneficial. We planned to explore the need for a PRISMA extension statement focused on maximizing the quality of reporting of network meta-analyses.

As part of the development of a statement extending PRISMA to cover network meta-analyses, we set out to examine the findings of the peer reviewed literature on the key limitations in the reporting of network meta-analyses. This literature review will help assess the need for reporting guidance, as well as focus the guideline on those features of most importance to clear reporting of network meta-analysis.

## Methods

A brief protocol was developed prior to initiation of this review. It can be acquired by request from the corresponding author.

### Ethics

No ethical approval was required for the performance of this work.

### Literature Search

An information specialist designed an electronic literature search to search for studies that assessed the quality of reporting of indirect treatment comparisons and network meta-analyses, as well as related key guidance documents for network meta-analysis. The search included a broad range of terms related to the concept of indirect treatment comparisons and network meta-analysis including *indirect treatment comparison*, *mixed treatment comparison*, *network meta-analysis*, and *multiple treatments meta-analysis*. The search was peer reviewed by members of the authorship team, as well as by an independent information specialist who employed the PRESS (Peer Review of Electronic Search Strategy) framework. [14] Medline and the Cochrane Methods databases were searched from January 2004-November 9, 2012, and an update was run in August 2013; the Medline search strategy is provided in an online supplement to this review (see Table S1).

### Inclusion Criteria and Study Selection

We included studies, in full text or abstract form, which assessed one or more aspects of the quality of reporting of network meta-analyses or indirect treatment comparisons. Relevant aspects of reporting included (but were not limited to) the following elements: completeness of literature search description; completeness and adequacy of reporting of statistical methods used for analysis; statement and description of assumptions made for network meta-analysis (common terminology including the words similarity, homogeneity, consistency, and transitivity [15]); adequacy of describing evidence included in the network of treatments; and completeness and adequacy of reporting of results from analysis (including summary estimates and related uncertainty, treatment rankings, probability-oriented parameters of interest, and strategies for presenting complete findings).

All citations identified from the literature search were screened independently by two individuals (BH, LT, FCL). Stage 1 screening used a liberal strategy where only one individual had to consider a citation of potential relevance in order for it to be advanced to Stage 2 full text screening. For Stage 2, the same individuals reviewed all potentially relevant works in full text. Final inclusion was confirmed if both individuals felt the study was directly relevant to the objectives of this review. Planned involvement of a third party to deal with unresolved discrepancies was not required.

### Data Collection

Two members of the team extracted data from each retrieved paper (BH, FCL). To enable description of the network meta-analyses reviewed in each study and the types of information collected, we gathered the following information from all studies: authorship list, inclusion criteria for types of meta-analyses, number of network meta-analyses reviewed, frequency of the various methods used, and primary authors’ results and conclusions drawn.

## Results

### Extent and Characteristics of Evidence Available from Past Reporting Quality Assessments

The literature search and recommendations from co-authors for additional papers identified a total of 775 citations for review. Stage 1 screening identified a total of 19 studies considered to be potentially relevant and whose full texts or supporting posters (where available) were obtained. Following Stage 2 screening, 9 of the studies were retained (6 full reports, 3 abstracts). The flow diagram in **Figure S1** presents the process of study selection.

The six full publications assessed indirect treatment comparisons [16] (n = 1) or a combination of indirect treatment comparisons and network meta-analyses [9], [15], [17]–[19] (n = 5) (one additional publication was excluded given its overlap with one of the other included reports [20]). One of the full reports [19] as well as three abstracts [21]–[23] reviewed the quality of conduct and reporting of indirect comparisons and network meta-analyses reported in health technology appraisals of the National Institute for Clinical Excellence (NICE) (the latter focused mainly on those conducted by pharmaceutical companies). All studies were published in 2009 or later. Table 1 provides a summary of the primary methodologic characteristics of the included studies, including inclusion criteria, types and size of indirect comparisons reviewed, data elements collected and conclusions of the study authors.

**Table 1:** Summary of Included Studies.

First Author(Year)	Cited Objectives	Inclusion Criteria	# of NMAs orITCs Included	Methodologic Components Reviewed	Authors’ Conclusions
**Included Full Reports**					
Nikolakopoulouet al (2014) [9]	Develop an understandingof characteristics of andproblems encountered inpast network meta-analyses.	Reports published prior to theend of 2012 that included anyform of indirect comparison;networks had to include aminimum of 4 treatments and adescription of data analysis.	186	Outcome, number of included studies, synthesismethod (e.g. Bucher method, hierarchical model,meta-regression), approach to evaluation of consistency, control treatment, number of competing interventions, network shape (e.g. star shape, closed loops), category of comparison (pharmacologic versus placebo, etc), outcome measure (e.g. odds ratio, risk ratio, etc)	While NMA validity is highly reliant on assumptions, the reporting and evaluation of these assumptions are commonly not addressed by authors
Bafeta et al[18] (2013)	To examine whether NMAsfollow the key methodologicalrecommendations for reportingand conduct of systematicreviews.	All NMAs from 2003-July 2012that compared clinical efficacyof three or more interventions basedon randomised controlled trials,excluding meta-analyses with anopen loop network of threeinterventions.	121	Reporting of general characteristics and keymethodological components of the systematic review process (e.g. PRISMA and AMSTAR) using twocomposite outcomes. For some components, ifreporting was adequate, the authors assessed theirconduct quality. Authors assessed whether NMAsmentioned or discussed the assumptions required(based on homogeneity, similarity, consistency, andexchangeability). Information regarding assessment of reporting bias and other details were also collected.	Essential methodological components of the systematic review process are frequently lacking in reports of NMAs, even when published in journals with high impact factors.
Tan et al[19] (2013)	To establish guidance and currentpractice on summarizing resultsfrom indirect comparisons andmixed treatment comparisons; to produce recommendations toimprove current practice; toidentify research priorities forimproving reporting.	HTA programme reports publishedbetween 1997–2011 by the NIHRin the UK that employed methodsfor indirect comparisons of mixedtreatment comparisons.	19 (8 indirect comparisons,11 mixed treatmentcomparisons)	Reporting of input data (presentation of the numberof interventions, study level data and the relationship structure of the interventions and the studies included in the analysis), methods (specification of Bayesian or frequentist statistical models, software used, presentation of prior distributions used, sensitivity analyses, model convergence assessment), results (presentation of relative and absolute effects, probability of treatment being best, ranking of interventions).	A variety of methods for reporting reviews involving these methods were identified. There is no current standard approach used. Standardization of reporting methods and improvement in the use of graphical approaches to present information are needed.
Coleman [17](2012)	Summarize guidance for NMA;summarize traits of publishedNMAs; understand reasons forchoice and implementation ofNMA	Studies from 2006– July 2011 wereincluded. Studies had to include 3or more treatments compared usingRCTs; published in full text;Bayesian or Frequentist approachaccepted; not included in a methodspublication or economic evaluation;English publication.	33 Bayesian (81%), 8frequentist, 1 both	Type of analysis (bayesian vs frequentist); reportingof methods choices (prior choice, inconsistencyevaluation, heterogeneity assessment, modelconvergence assessment); number of treatments;number of trials and patients; funding and country;clinical area; network pattern; involvement of amethodologist; fixed or random effects; use ofcovariate adjustments; account for multi-arm trials;software used; reporting details (type of outcome,summary measure, probabilities and claims ofequivalence or non-inferiority.	Further guidance on proper conduct, interpretation and reporting of network meta-analyses is required.
Donegan [16](2010)	Review quality of publishedindirect treatment comparisons to add to empirical data supporting a need for improvements ofreporting of such work.	**Inclusion**: reviews applying statistical methods to indirectly compare effects of 2 interventions based on RCTs. A statistical method was considered to be a quantitative summary of the ITC or a description of overlap of the two related CIs. **Exclusion**: ITCs shown forillustrative purposes; ITCs based onnon-randomized data; costeffectiveness and narrative reviews;trial reports; qualitative ITCs;MTC/NMA reviews.	43 ITCs (review excluded NMAs); all based on frequentist methods	Study inclusion criteria; ITCs made; clinical area ofstudy; # of trials and patients involved in ITC; thetype of data and measure of effect; consideration &assessment of similarity, homogeneity and consistency(with detailed criteria for each); approach to reportingof results and interpretation. Criteria based on theabove components used to identify higher quality andlower quality ITCs.	The assumptions of ITCs aren’t always explored or reported. Reporting should be improved by more routine assessment of assumptions and clear statement of methods used for assessments.
Song [15](2009)	Assess the basic assumptions and other current limitations in the use of indirect comparisons in systematic reviews of healthcareinterventions	Systematic reviews/meta-analysespublished between 2007–2007which used an indirect comparison(based on title/abstract).	88 indirect comparisons (49/88 frequentist adjusted ITC, 18/88 NMA or Bayesian MTC, 13/88 informal ITC, 6/88 naive ITC, 2/88 unclear)	Collected data related to comprehensiveness ofliterature search for trials included in indirectcomparisons, methods used to conduct indirectcomparison, availability of head-to-head trial data,presence/absence of explicit mention of the similarityassumption (and related efforts to explore/improvesimilarity)	Several key methodologic problems related to authors’ unclear understanding of key assumptions for ITC. Sub-optimal search for evidence, statement and exploration/resolution of trial similarity and inappropriate combination of direct and indirect evidence are all current problems.
**Included Abstracts**					
Brooks-Renney[23] (2011; abstract)	Review the use of NMAs within published health technology appraisals from NICE.	Technology appraisals from NICEpublished between 2006–2011containing the term ‘mixed treatment comparison’. Submissions which werewithdrawn, terminated or suspended were not included.	17 included technology appraisals	Specific criteria gathered are not described. Providesinformation related to methodologic limitationsidentified in the conduct of network meta-analysessubmitted by manufacturers.	NMA and ITC are increasingly common in technology assessments, however robust design and methdologies are needed to enesure maximum uptake of their findings.
Bending [21](2011; abstract)	Review the methodology andimpact of ITCs and NMAssubmitted by manufacturerson the NICE committee’sappraisal of pharmaceuticals.	Technology appraisals from NICEpublished between 2006–2011containing either of the terms‘indirect treatment comparison’or ‘mixed treatment comparison’.Submissions which were withdrawn,terminated or suspended were notincluded.	24 included technologyappraisals	Number of trials included, availability of head-headevidence, disease area, treatment comparisons made,justification of study selection, sensitivity analysesrelated to trial selection, outcomes assessed. Alsocollected related information on key critiques of ITCsand NMAs.	There is wide variation in the reporting and validity of ITCs and NMAs. There is a need for guidance for the conduct of NMAs and ITCs.
Buckley [22] (2009 abstract)	To investigate factors impactingoutcome of health technologyassessment submissions involvingindirect and mixed treatmentcomparisons.	Technology appraisals from NICEpublished between 2003–2008	19 published technologyappraisals	Collection of evidence presented in HTA submissionsincluding therapeutic area, clinical comparisons made,degree of direct and indirect evidence, suitability andcriticisms of type of analysis and statisticalmethodologies used, outcome appraisal (full/partialrecommendation versus not recommended)	There is a clear increase and acceptance in use of indirect comparison methods in technology assessments. To maximize their quality, past criticsms related to use of validated methods, proper accounting for population heterogeneity, clear justification of analysis decisions made need to be considered.

Narrative descriptions of the findings from the included papers have been stratified below according to the type of publication (full versus abstract) in which the research was described. **Table 1** provides an overview of the methods and findings of studies included in this review.

### Full Reviews of Reporting Quality Published Indirect Comparisons and Network Meta-Analyses

Bafeta et al [18] (2013) conducted a systematic review of published network meta-analyses comparing the clinical efficacy of three or more interventions based on RCTs, excluding reviews with three interventions and no closed loop of evidence. The authors examined the reporting of general characteristics and key methodological components of the systematic review process. A total of 121 network meta-analyses from an assortment of clinical disciplines (e.g. Cardiology 22.3%, Rheumatology 13.2%, Endocrinology 9.9%, Oncology 7.4%) published prior to July 2012 were included, and 100 (82.6%) described the assessment of pharmacological interventions. Regarding the reporting of study methods, the electronic search was not reported in 72.7% (88/121) of reports, and there was no clear specification of the primary outcome in 29.7% (36/121) of reports. Totals of 34.7% (42/121) and 26.4% (32/121) did not report the methods used for study selection and data extraction, respectively. Overall, 50.4% 61 NMAs (50.4%) did not report any information regarding the assessment of risk of bias of individual studies, and 103 (85.1%) did not report methods for the assessment of publication bias. Regarding the reporting of results, 95 NMAs (78.5%) did not describe the characteristics of primary studies (e.g. characteristics of the network, patient characteristics and interventions), while 70 (58.5%) did not report findings from risk of bias assessments of the included studies. Regarding the underlying assumptions for network meta-analysis, the similarity and consistency assumptions were not explicitly mentioned in totals of 66.1%(80/121) and 52.1% (63/121) of the included studies. Bafeta et al concluded that key methodological components of the systematic review process are frequently inadequately reported in published NMAs, and that this inadequate reporting of NMAs raises doubts about their ability to help determine the best available treatment in comparative effectiveness research.

Nikolakopoulou et al [9] reported descriptive information from a review of 186 network meta-analyses published prior to 2013 which included networks comparing a minimum of four treatments, with the objective of developing insights on characteristics of networks in health research. A total of 35 networks were star-shaped (i.e. active interventions were compared against a common treatment but not against one another), while a median of 6 treatments (IQR 5–9 treatments) were compared and a median of 21 studies (IQR 13–40 studies) were synthesized. A total of 113/186 (61%) of the meta-analyses were performed using a Bayesian approach, while in 18 papers the approach to synthesis was not clearly reported. About two thirds (60%) of the analyses studied a dichotomous outcome measure, while continuous (28%), time-to-event (9%) and rate (3%) outcomes were less common. With regard to aspects of reporting of NMAs, the authors noted several points of concern. Regarding approach to analysis, it was noted that 9 (26%) of the 35 included star-shaped networks failed to specify their approach to data analysis, as did 9 (6%) of the other 151 included networks. The authors noted there is no sign this aspect of reporting has improved with time, as totals of 11% of 2007 NMAs, 5% of 2011 NMAs and 8% of 2012 NMAs fell into this category. Regarding inconsistency, the authors observed that over time an increased number of authors have realized the importance of the consistency assumption and have reported their efforts and findings to address this issue. Overall, the authors concluded that while reviews involving NMA may use appropriate methodology, readers’ dependence upon the reporting of the methods used could impact the study’s conclusions, and guidance for reporting of NMAs is needed.

Tan et al [19] (2013) reviewed reports published between 1997 and 2011 in the UK Health Technology Assessment (HTA) programme which considered indirect and/or mixed-treatment comparison methods with respect to the presentation of methods and results. The authors also reviewed existing institutional guidance and developed recommendations for presentation. Of 205 HTA reports that contained evidence syntheses, 19 used indirect comparisons (n = 8) and/or mixed-treatment comparison methods (n = 11), respectively. All 19 reports were published after 2004, the year in which NICE guidance recommended the use of indirect comparisons analysis when no head-to-head RCTs exist. Overall, a high variability was shown in the presentational formats from which some key components were identified (e.g. network diagrams or tables, model descriptions to allow reproducibility, and tables and forest plots for presenting a range of results). The authors concluded that standardization of reporting and innovation in graphical representation of indirect and/or mixed-treatment comparisons is required.

Coleman et al [17] (2012) published findings from a study whose objectives included summarizing existing guidance for network meta-analysis, as well as summarizing characteristics of published NMAs including aspects of reporting quality. In the current review, findings from only the second objective are discussed. The authors studied the characteristics and reporting of a total of 43 network meta-analyses; characteristics of reviews using Bayesian versus Frequentist approaches to evidence synthesis were reported separately. Bayesian network meta-analyses were cited as having several limitations related to reporting, one being a failure to provide adequate description of the statistical methods used (e.g. failure to report on use of adjustments for multi-arm trials, failure to adequately describe the prior distributions used, failure to describe assessment of model convergence, failure to report on model fit assessment). Near 70% of Bayesian analyses assessed the assumptions underlying network meta-analysis using an assortment of methods that included comparison with estimates from traditional meta-analysis, study of inconsistency factors, review of posterior residual mean deviance, and reporting of inconsistency variance. Regarding reporting of results, most reviews (32/34 = 94.1%) included some findings in the main text, while many also presented a summary of results in tabular form (24/34 = 70.5%) or a figure (21/23 = 61.8%). Beyond relative effect measures, 61.8% also reported a ranked order of treatments based on estimated probabilities of superiority. Few studies were found to have indicated whether study point estimates were mean or median summary values, and few studies (21/34 = 61.8%) provided access to raw data. From the nine included articles that employed Frequentist network meta-analyses, the authors noted limitations regarding use of a different terminologies to describe the method to analysis (including “frequentist framework using random effects”, “mixed effects hierarchical model with a log link function”, “random effects non-linear mixed model based on pseudolikelihood”, “online program published by Lumley”, and “frequentist mixed effects meta-regression”), a failure to describe weighting of studies (8/9 = 88.9), and a failure to indicate whether or not covariate adjustments were performed (7/9 = 77.8%). Based on their review, the authors concluded there is a clear need for further guidance on optimal reporting of network meta-analyses.

Donegan et al [16] (2010) completed a systematic review of published reviews involving indirect treatment comparisons (but excluding network meta-analyses) to explore reporting limitations. Comparisons in this research could be based on an adjusted indirect approach [4], though description of the overlap of two confidence intervals for competing treatments of interest relative to a common comparator were also included. The authors developed a list of criteria against which to assess included reviews: items were related to the mention, description and assessment of the key assumptions of network meta-analysis, and how results were reported and interpreted. Overall 43 published indirect comparisons published between 1992 and 2007 were included. Regarding specific elements of quality evaluation, the following observations were noted: 11/43 (25.6%) explicitly stated the similarity assumption, while 0/43 stated how it would be empirically assessed; 38/43 (88.4%) reported study and patient characteristics in the manuscript, and 19/43 (44.2%) assessed similarity via meta-regression, subgroup analysis or sensitivity analysis. A total of 11/43 (25.6%) compared trial level characteristics across studies, however only 4/11 were described as comparable, 5/11 were not, and 2/11 were unclear. Regarding the homogeneity assumption, 24/43 (55.8%) assessed statistical homogeneity, and 12/43 (27.9%) assessed causes of statistical heterogeneity. Regarding consistency, 17 reviews included both direct and indirect evidence, however a total of only 6 (35.3%) assessed consistency. With regard to reviews’ discussion and conclusions, 25/43 (58.1%) of them urged caution in the interpretation of results, and 24 (55.8) indicated when results were based on indirect evidence. Donegan et al concluded that the underlying assumptions of indirect comparisons are not always explored or reported by researchers, and that reporting should be improved by more routine assessment of assumptions and by clear statement of methods used for these assessments.

Song et al [15] (2009) reviewed 88 systematic reviews published between 2000 and 2007 that involved the estimation of an indirect comparison in the form of either an adjusted indirect comparison, a frequentist/bayesian network meta-analysis, an informal indirect comparison, or a naive indirect comparison. These reviews came in various formats including journal-based reviews of effectiveness (n = 59), technology assessment/cost effectiveness studies (n = 19), Cochrane reviews (n = 6), and methods articles illustrating aspects of the conduct of indirect comparisons (n = 4). The authors reported several deficiencies from this collection of reviews, including a predominantly unclear understanding of authors of the assumptions for indirect comparisons, inappropriate search for and selection of trials, lack of sound means to assess similarity of trials and/or efforts to improve it, and inadequate comparison of direct and indirect evidence (often leading to an inappropriate combination thereof). Recommendations were made to improve the description and discussion of the key underlying assumptions for indirect comparisons, to achieve more systematic literature searches for evidence, to rationalize exclusions of head-head evidence, to appropriately account for multi-arm studies, to better compare direct and indirect evidence before combination thereof, and to only combine direct and indirect evidence after such explorations warrant doing so.

### Eligible Abstracts Describing Network Meta-Analyses from Technology Assessments

Three studies described in recent abstracts[21]–[23] explored the quality of network meta-analyses and indirect treatment comparisons submitted by drug manufacturers to the National Institute of Clinical Excellence between 2006 and 2011. Brooks Renney et al [23] reviewed a total of 17 network meta-analyses (15 from pharmaceutical companies, 2 performed by the National Institute for Clinical Excellence [NICE] review group) that spanned a range of clinical disciplines and collected information regarding limitations that were noted by the review team. Overall, 12/15 (80%) of manufacturer-submitted analyses were suggested by the appraisal committee as needing to be interpreted with caution due to inappropriate inclusion or exclusion of clinical studies, inadequate detail regarding the statistical approach used, insufficient appraisal of heterogeneity that existed between studies, and inappropriate use of subgroup data rather than complete study population data. In similar work, Bending et al [21] reviewed 24 reports submitted to NICE that contained either an indirect treatment comparison or a network meta-analysis. The authors found 18/24 (75%) reports to have validity issues. Key problems noted were a lack of reporting of trial characteristics, a lack of description of methods for trial quality assessment, inappropriate methods of analysis, inappropriate exclusion of trials, and a presence of clinical and statistical heterogeneity between included trials. Buckley et al [22] also reviewed NICE technology appraisals from 2003–2008, and found the key limitations noted in these appraisals were related to limitations for justification of methods used, assumptions made, treatment comparators chosen, and failure to adequately deal with clinical and statistical heterogeneity.

### Summary of Themes Identified from this Review

Based on findings from the included reviews and subsequent discussion amongst co-authors, we categorized the perceived key elements related to reporting of both methodology and findings which were judged most important for consideration in a future reporting guidance document. These elements are summarized in **Table 2** (methodology) and **Table 3** (results), respectively. This information has played an important role thus far in the conduct of a survey to gather opinions from systematic reviewers on reporting of network meta-analyses, and will also be vital in the future development of a PRISMA extension statement specific to network meta-analysis.

**Table 2:** Summary of Considerations for Methodologic Reporting.

**Literature Search:**
• Specification of efforts to search for direct and indirect evidence of relevance
• Presentation of search terms and the full search strategy (or strategies if separate searches undertakenfor each comparison) for one electronic database
• Provision of information regarding involvement of primary literature searching versus use of existingsystematic reviews as a means of including studies
• If existing reviews were used, specification of how these were located and description of theirrelated inclusion criteria
**Specification of Eligibility Criteria (and Planned Network Structure):**
• Specification of PICOS eligibility criteria for the review, including specification of all treatmentsincluded in the planned meta-analysis
• Specification of how related but different implementations of the same agent (e.g. varied doses ofpharmacologic treatments) are to be handled with associated rat ionale (i.e. address ‘lumping and splitting’of interventions)
**Specification of Assumptions for Network Meta-Analysis:**
• Specification of the assumptions of homogeneity, similarity and consistency (or related terminologyused, e.g. transitivity, exchangeability)
• Specification of efforts taken by reviewers to evaluate the assumption appropriateness
• Specification of what information is being provided to readers to allow them to consider the validityof the assumptions
**Statistical Approach to Analysis:**
• Specification of details of the approach to statistical analysis taken: hierarchical model, adjustedindirect approach or meta-regression, Frequentist or Bayesian framework, fixed or random effectsmodel, homogeneous or heterogeneous between-trial variance structure
• Specification of methods used to assess the degree of statistical heterogeneity and the potential forpublication bias within the treatment network
• Specification of methods used to evaluate for the presence of disagreement between direct and indirectevidence in the treatment network
• Description of statistical methods used to address clinical and methodologic homogeneity in theanalyses (e.g. subgroups, meta-regression including adjustments for baseline risk and the impactof risk of bias variations)

**Table 3:** Summary of Considerations for Reporting and Interpretation of Results.

**Presentation of Evidence and its Characteristics in the Treatment Network**
• Presentation of network diagram to summarize identified evidence
• Reporting information reflecting the amount of information in the network, e.g. sample sizes,numbers of studies per comparison and the presence of multi-arm studies
• Presentation of information allowing readers to assess clinical and methodological heterogeneitywithin the treatment network: e.g. information tables listing effect modifiers across studies andcomparisons, These can include patient characteristics and risk of bias assessements
**Assessment of Assumptions for Network Meta-Analysis**
• Information to summarize evaluations of statistical heterogeneity within the treatment network
• Information and approach to summarize analyses to assess agreement of direct and indirectsources of evidence (and efforts to improve agreement if discrepancies are found)
**Presentation of Summary Treatment Effect Estimates and Related Measures**
• What estimates to report: all possible pairwise comparisons? Only those which are comparisonsagainst the chosen reference group or a treatment of primary focus?
• Should findings from traditional pairwise analyses also be provided?
• Presentation of summary estimates and corresponding uncertainty (i.e. credible/confidence intervals)
• Presentation of summary estimates from sensitivity and subgroup analyses
• Optimal use of tables and figures to most easily convey results to readers
• Presentation of treatment rankings and corresponding probabilities: Should they be included?What should be presented?
**Discussion and Conclusions**
• Commentary on the clinical and biologic plausibility of the observed findings
• Commentary relevant to any important concerns regarding the assumptions underlying thejoint synthesis that may play an important role in strength of interpretations drawn

## Discussion

Network meta-analysis represents an increasingly prevalent extension of traditional meta-analysis. In addition to continued training needs, our findings suggest a need to develop and promote guidance for reporting these types of studies to maximize transparency and replicability, a corner stone of all science. Such guidance will benefit systematic reviewers with limited exposure to network meta-analyses. Current guidance from groups associated with NICE[12], [24]–[26] and ISPOR [10], [11] largely provide insights on conduct and interpretation. The 2009 PRISMA statement [1] provided researchers with a delineation of core elements to maximize the transparency and completeness of reporting for pairwise systematic reviews with meta-analyses; we conducted this overview to identify what items may be needed for an extension statement to address reporting of network meta-analyses. In addition to producers of clinical research, these tools will also prove fruitful for those faced with peer review of network meta-analyses who may require a framework to guide their evaluations. Technology assessment agencies and pharmaceutical and device manufacturers will also benefit from such guidance, which will provide clarity as to how network meta-analyses and indirect treatment comparisons could be more clearly reported.

This overview identified a total of nine publications which included some form of assessment of the reporting quality of past network meta-analyses or indirect treatment comparisons. This data has identified several limitations for which reporting guidance could be generated to improve reporting. These components included several aspects related to the replicability and validity assessment of NMAs, important aspects of the approach to data analysis, and the reporting of findings. While not meeting our inclusion criteria, we also reviewed a recent report by Lee [8] which noted a need for improved tagging of such studies in literature databases, mixed used of terminologies across reports, and which also made general reference to mixed degree of detail regarding aspects of network meta-analysis; all of these cited limitations can be at least partially improved with better reporting. We also reviewed a number of guidance documents published between 2005–2012 from both the peer reviewed literature and from technology assessment agencies, [6], [7], [10]–[12], [24]–[39] and found that these works reinforced the importance of the items that we found to be current limitations in the literature based on the included assessments of reporting quality. While we did not identify emphasis on the reporting of absolute measures of risk, this represents an additional reporting consideration for discussion to meet the needs of decision makers.

There are limitations to note for this review. First, our sampling frame was not extant, which would require a primary search for and risk of bias evaluation of all such studies. In our view, conducting an overview provides an adequate rationale for the need to consider extending the PRISMA statement for reporting network meta-analyses. Second, we did not perform a comprehensive search of the literature to identify all methodologic articles related to the conduct of network meta-analysis, which could also serve as a source of topics possibly worthy of reporting guidance. We relied upon the expertise and experience of our authorship team to identify additional items for inclusion in a Delphi survey exercise which was implemented during the summer of 2013, and the survey participants were also provided the opportunity to do so in the context of the survey. Third, we did not perform a risk of bias assessment of the studies included in this report. However, as our primary objective was to compile a list of potential checklist items for inclusion in the development of reporting guidance for network meta-analysis, this did not detract from achievement of our goal.

## Conclusions

Currently available literature regarding the reporting of network meta-analyses is sparse. Based on the existing evidence, several deficiencies in the reporting of network meta-analyses are apparent and we believe extending the PRISMA statement to network meta-analyses is the best resolution. This overview provided an excellent basis for a Delphi panel survey held in the summer of 2013 and a subsequent face-to-face meeting of experts in the fall of 2013. Dissemination of work generated from this process will be pursued in the near future.

## Supporting Information

Figure S1 **The flow diagram for study selection.**
(TIF)

Table S1 **The Medline search strategy used for this syetmatic review.**
(DOCX)

Checklist S1 **presents a PRISMA checklist assessment for this review article.**
(DOC)

Protocol S1 (DOCX)
