# Supplementary figures and images for: The Quality of Reporting Methods and Results in Network Meta-Analyses: An Overview of Reviews and Suggestions for Improvement
Source: PLoS One. 2014 Mar 26;9(3):e92508. doi: 10.1371/journal.pone.0092508 (PMC3966807; doi:10.1371/journal.pone.0092508)

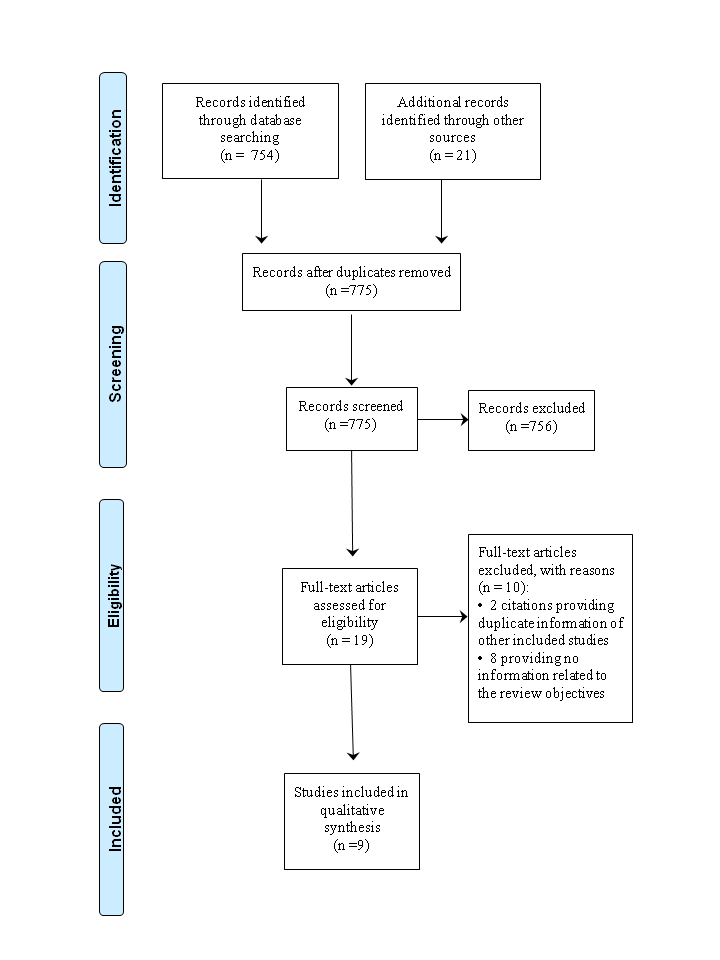

Supplement: Figure S1 — The flow diagram for study selection. (TIF) [file pone.0092508.s001.tif]
