# Supplementary material for: The Quality of Reporting Methods and Results in Network Meta-Analyses: An Overview of Reviews and Suggestions for Improvement
Source: PLoS One. 2014 Mar 26;9(3):e92508. doi: 10.1371/journal.pone.0092508 (PMC3966807; doi:10.1371/journal.pone.0092508)
Supplement: Table S1 — The Medline search strategy used for this syetmatic review. (DOCX) [file pone.0092508.s002.docx]

**Online Supplement, Literature Search Strategy**

Database: Embase Classic+Embase <1947 to 2012 July 20>, Ovid MEDLINE(R) In-Process & Other Non-Indexed Citations and Ovid MEDLINE(R) <1946 to Present> Search Strategy:

--------------------------------------------------------------------------------

1 ((network* or network-based) adj3 (meta-analy* or metanaly* or metaanaly* or met analy*)).tw

2 (MTC adj3 (meta-analy* or metanaly* or metaanaly* or met analy*)).tw. (37)

3 ((mixed treatment* or multiple treatment*) adj3 (compar* or meta-analy* or metanaly* or metaanaly* or met analy*)).tw

4 (Indirect* adj2 compar*).tw

5 or/1-4

6 exp Quality Control/

7 Publishing/

8 Publication Bias/

9 Research Report/

10 Periodicals as Topic/

11 Checklist/

12 (mt or st).fs

13 evaluation studies.pt

14 ((report* or method* or publicat*) adj5 (assess or apprais* or bias* or characteristic* or criteri* or critiqu* or evaluat* or quality or checklist* or check list* or score$1 or scoring)).tw

15 (reporting or methodolog*).tw

16 or/6-15

17 5 and 16

18 17 use prmz

19 ((network* or network-based) adj3 (meta-analy* or metanaly* or metaanaly* or met analy*)).tw

20 (MTC adj3 (meta-analy* or metanaly* or metaanaly* or met analy*)).tw. (37)

21 ((mixed treatment* or multiple treatment*) adj3 (compar* or meta-analy* or metanaly* or metaanaly* or met analy*)).tw

22 (Indirect* adj2 compar*).tw

23 or/19-22

24 exp quality control/

25 exp publishing/

26 exp evaluation research/

27 exp checklist/

28 ((report* or method* or publicat*) adj5 (assess or apprais* or bias* or characteristic* or criteri* or critiqu* or evaluat* or quality or checklist* or check list* or score$1 or scoring)).tw.

29 (reporting or methodolog*).tw

30 or/24-29

31 23 and 30

32 31 use emczd

33 18 or 32

34 limit 33 to yr="2003-current"

35 remove duplicates from 34

36 35 use prmz

37 35 use emczd
