# Supplementary material for: The Quality of Reporting Methods and Results in Network Meta-Analyses: An Overview of Reviews and Suggestions for Improvement
Source: PLoS One. 2014 Mar 26;9(3):e92508. doi: 10.1371/journal.pone.0092508 (PMC3966807; doi:10.1371/journal.pone.0092508)
Supplement: Protocol S1 — (DOCX) [file pone.0092508.s004.docx]

**Review Protocol:**

**Overview of Reviews of Reporting Quality in Network Meta-Analyses**

Prepared by: Brian Hutton (Ottawa Hospital Research Institute)

***Brief description.*** This document provides rationale and a brief summary of plans to perform an overview of reviews that have assessed one or more aspects of reporting quality issues in published network meta-analyses/mixed treatment comparisons/indirect treatment comparisons. This work is being performed as part of a process planned to develop reporting guidelines in the form of an extension of the PRISMA statement for network meta-analysis. This work will be used to develop a delphi survey that will be implemented to collect opinions from systematic reviewers and users of network meta-analysis to construct this guidance.

**Background**

Systematic reviews incorporating meta-analyses (SRMA) have long been used to derive summary comparison measures from multiple sources of evidence, most commonly randomized clinical trials (RCTs), to establish the effectiveness and safety of one treatment relative to another. This approach to evidence synthesis is a standard in evidence based medicine. SRMAs have long been considered a rigorous means of comparing pairs of different medical interventions. To maximize their transparency, methodologic quality and consistency of reporting, the Quality of Reporting of Meta-Analyses (QUOROM) checklist for authors was developed in 1999. In 2009, the Preferred Reporting Items for Systematic reviews and Meta-Analysis (PRISMA) statement was developed as a robust update to QUOROM to cover subsequently noted items that were considered essential.

Over time, increasingly large numbers of treatments for many medical conditions have provided clinicians with more choices from which to select a treatment strategy for their patients. Regulators have only required evidence of benefit over no treatment and a lack of evidence of harms for approval to market. The resulting absence of motivation for drug developers to compare their products against those of their competitors has promoted analytic methods to establish the relative benefits of new agents relative to existing therapies. Bucher et al proposed the adjusted indirect comparison, and in subsequent years Lu and Ades (2004) described methods for network meta-analysis to simultaneously compare a network of treatments, subsets of which have been compared in individual studies.

The frequency of use of network meta-analysis has risen notably since the mid 2000s. A rapid evolution of methods for NMA has raised concerns that the conduct and reporting of network meta-analyses may not yet be adequate. The PRISMA statement was developed to provide systematic reviewers with guidance on elements to produce optimal reporting of systematic reviews and meta-analyses of pairwise treatment comparisons in order to maximize the transparency, replicability, and quality of such studies. Compared to pairwise comparisons of two treatments, network meta-analysis requires more complex meta-analytic techniques that are associated with additional assumptions, a more complex statistical framework, and generates additional outputs of potential interest to readers (for example, treatment ranks and corresponding probabilities) which can complicate the presentation of results. While documents providing guidance for systematic reviewers and readers on conduct and interpretation of network meta-analysis are available, a guidance document for reporting may also be beneficial. We intend to develop an extension of the PRISMA statement for network meta-analysis, and a review identifying reporting limitations of current NMAs will be fruitful to help inform this initiative. This literature review will help assess the need for reporting guidance, as well as focus the guideline on those features of most importance to clear reporting of network meta-analysis.

**Literature Search**

The final literature search strategy is provided in the appendix to this protocol and was developed by an information specialist after she was provided with key seed terminology upon which to develop the search. Appropriate date ranges covering Medline/Pubmed and the Cochrane methods database will be chosen by the review librarian (Mrs. Rebecca Skidmore) at the time the search is run. An updated search will be performed when nearing completion of the review to look for any new and important reports meeting our eligibility criteria that may have recently been published. Searches will use the OVID interface.

**Screening and Data Collection**

- Stage 1 screening of abstracts and Stage 2 screening of full text articles will be performed independently by two reviewers; where needed, a third individual will be consulted to break disagreements regarding final inclusion status of manuscripts.
  - Synopsis, inclusion criteria: we will seek reviews which have in some manner, whether a primary or secondary objective, provided data which in some way relates to any aspect of the quality of reporting of systematic reviews incorporating network meta-analyses. Aspects of reporting can include rationale for using NMA, methodologic considerations to ensure transparency of methods (e.g. network geometry choice, statistical methods, assumptions for NMA, etc), and approaches/transparency to summarizing results from NMAs performed.
  - Full publications and abstracts are both eligible. Only studies published in English will be eligible for inclusion.
  - The process of study selection will be reported using a PRISMA flow diagram.
- Data collection will also be performed by two reviewers. As the objective of this review is primarily to cover any and all information from existing systematic reviews somehow reporting an assessment deemed related to reporting quality, the approach to data collection will be relatively open.
  - Information we will collect from included manuscripts will include authorship and publication-related information; the number of networks/indirect comparisons assessed in each of the included systematic reviews; the objective of each study; the inclusion criteria of each study; the aspects of reporting quality addressed by each study (e.g. specifics about reporting of methods of NMAs including definition of network structure and statistical methods, approaches to reporting of summary estimates and related parameters summarizing treatment effectiveness/safety, and so forth); the conclusions drawn by the study authors.
- Data collectors will meet prior to the start of data collection to discuss the planned approach to description of included studies to ensure sufficient information is gathered to allow for a sufficiently detailed narrative summary of each study.

**Data Analysis**

- No quantitative data analysis is planned for this overview.
- The overview will be narrative in nature in order to provide readers with a clear understanding of the methods and findings of each included review, as well as the findings/conclusions from each team of authors.
- The main objective of this review is to establish if existing work in this area supports the need for reporting guidance related to network meta-analyses. In addition, an important objective is to identify aspects of network meta-analyses which currently appear to be associated with the most common reporting challenges to ensure these are later addressed in the planned development of reporting guidance for NMA.
- We will use a combination of tables/figures as judged needed to summarize study characteristics, present key findings, and identify author conclusions with regard to key messages related to the reporting of network meta-analyses.

**Scope of Work**

- We will seek publication of this work to summarize our findings in the future.
- The primary goals of conducting this review are to (1) identify key elements for inclusion in reporting guidance for network meta-analyses; (2) establish key aspects related to reporting of network meta-analyses which require further discussion/opinions from key individuals in order to achieve consensus amongst experts and then subsequently develop helpful reporting guidance for network meta-analysis. This input will be sought through both a delphi survey to be implemented online and a subsequent face to face meeting to address particular items/topics upon which participating experts remain ‘at odds’.
- Following completion of this review, we will also look at key existing guidance manuscripts from the literature related to NMA to see whether any additional important considerations emerge which were not identified by our review.

**Appendix: Literature Search Strategy**

Database: Embase Classic+Embase <1947 to 2012 July 20>, Ovid MEDLINE(R) In-Process & Other Non-Indexed Citations and Ovid MEDLINE(R) <1946 to Present> Search Strategy:

--------------------------------------------------------------------------------

1 ((network* or network-based) adj3 (meta-analy* or metanaly* or metaanaly* or met analy*)).tw

2 (MTC adj3 (meta-analy* or metanaly* or metaanaly* or met analy*)).tw. (37)

3 ((mixed treatment* or multiple treatment*) adj3 (compar* or meta-analy* or metanaly* or metaanaly* or met analy*)).tw

4 (Indirect* adj2 compar*).tw

5 or/1-4

6 exp Quality Control/

7 Publishing/

8 Publication Bias/

9 Research Report/

10 Periodicals as Topic/

11 Checklist/

12 (mt or st).fs

13 evaluation studies.pt

14 ((report* or method* or publicat*) adj5 (assess or apprais* or bias* or characteristic* or criteri* or critiqu* or evaluat* or quality or checklist* or check list* or score$1 or scoring)).tw

15 (reporting or methodolog*).tw

16 or/6-15

17 5 and 16

18 17 use prmz

19 ((network* or network-based) adj3 (meta-analy* or metanaly* or metaanaly* or met analy*)).tw

20 (MTC adj3 (meta-analy* or metanaly* or metaanaly* or met analy*)).tw. (37)

21 ((mixed treatment* or multiple treatment*) adj3 (compar* or meta-analy* or metanaly* or metaanaly* or met analy*)).tw

22 (Indirect* adj2 compar*).tw

23 or/19-22

24 exp quality control/

25 exp publishing/

26 exp evaluation research/

27 exp checklist/

28 ((report* or method* or publicat*) adj5 (assess or apprais* or bias* or characteristic* or criteri* or critiqu* or evaluat* or quality or checklist* or check list* or score$1 or scoring)).tw.

29 (reporting or methodolog*).tw

30 or/24-29

31 23 and 30

32 31 use emczd

33 18 or 32

34 limit 33 to yr="2003-current"

35 remove duplicates from 34

36 35 use prmz

37 35 use emczd
